# Developing Smartness in Emerging Environments and Applications with a Focus on the Internet of Things

**DOI:** 10.3390/s22228939

**Published:** 2022-11-18

**Authors:** Rashid Mehmood, Juan M. Corchado, Tan Yigitcanlar

**Affiliations:** 1 High Performance Computing Center, King Abdulaziz University, Jeddah 21589, Saudi Arabia; 2 Bisite Research Group, University of Salamanca, 37007 Salamanca, Spain; 3 Air Institute, IoT Digital Innovation Hub, 37188 Salamanca, Spain; 4 Department of Electronics, Information and Communication, Faculty of Engineering, Osaka Institute of Technology, Osaka 535-8585, Japan; 5 City 4.0 Lab, School of Architecture and Built Environment, Queensland University of Technology, 2 George Street, Brisbane, QLD 4000, Australia

## 1. Introduction

The smartness that underpins smart cities and societies is defined by our ability to engage with our environments, analyze them, and make decisions, all in a timely manner [1]. We are witnessing a rapid evolution, or rather, a transformation of our societies. Novel solutions are being developed and adopted in work and life, benefiting from the growing ability to monitor and analyze our environments in near real time. A range of devices and technologies are being used for monitoring purposes, including the Internet of Things (IoT), the Global Positioning System (GPS), sensors, cameras, Radio Frequency Identification (RFID) devices, smartphones, smartwatches, other smart wearables, and social media platforms. These devices produce diverse data that are analyzed using Artificial Intelligence (AI) and other computational intelligence methods and are used for decision-making purposes. While significant advances have been made in developing smart applications and technologies, a systematic effort to define and develop “smartness” is missing. An investigation into the theoretical and technological foundations of this “smartness” can help systemize and mass-produce technologies for autonomous production and for the operation of smart environments.

**Figure 1:**
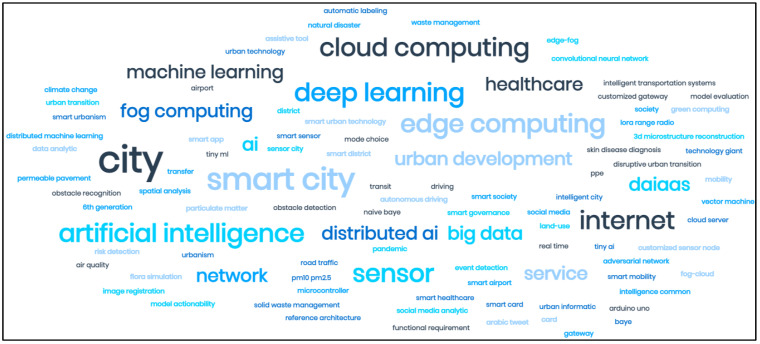
A Word Cloud of the Research Topics in this Special Issue.

This Special Issue’s focus is on the IoT, and it is concerned with bringing “smartness” to the IoT and other system layers using technologies such as Cloud, Fog, and Edge Computing; High-Performance Computing (HPC); Big Data; Blockchain; and/or AI. In addition to this Editorial piece, a collection of 13 articles is featured in this Special Issue, covering a range of topics, including mobility, healthcare, image analysis, permeable pavements, solid waste management, sensor node and gateway architectures, air quality monitoring, thermal anomalies and smart helmets in industrial environments, smart airports, smart districts, smart travel choices, sensor cities, artificially intelligent cities, and platform urbanism. Figure 1 provides a word cloud which represents the themes explored by this Special Issue.

Smartness is a multidisciplinary topic and can be defined from different perspectives. We see through the articles included in this Special Issue that smartness can be seen to have four dimensions (however, this is not the only way to look at it). These dimensions are: (i) Sensors, IoT, and Data Generation; (ii) Data and Information Processing; (iii) Actuation; and (iv) Digital Systems and Infrastructure. To elaborate, we can see smartness in the way sensing is embedded in a system, the way data and information are processed, how a system interacts internally and with its environment, and whether a system is ubiquitous or limited by space (cloud-based or edge-enabled). What follows is a brief review of the articles included in this Special Issue, which highlights their contributions with respect to these four dimensions. They are grouped according to their application areas: mobility and transportation, healthcare, industrial environments, and other urban infrastructures.

## 2. Mobility and Transportation

Transportation is the backbone of modern economies, albeit at massive human, environmental, and economic costs [2,3]. Lana et al. [4] assess that advances in data science have permeated into every field of transportation science and engineering, resulting in data-driven transportation developments. The authors describe how data from various intelligent transport system (ITS) sources can be used to learn and adapt data-driven models for the efficient operation of ITS assets, systems, and processes and how data-based models can become fully actionable. Furthermore, the authors define the characteristics, engineering requirements, and challenges inherent to the three compounding stages, namely, data fusion, adaptive learning, and model evaluation, based on the described data modelling pipeline for ITS. This work’s theoretical framework contributes to the first three dimensions of smartness, namely, sensors, data processing, and actuation.

Chia et al. [5] use smart card data to investigate the relationship between the spatial distribution of relative transfer locations and the attractiveness of the transit service. Transfers are an important part of transit trips because they enable people to reach more destinations; however, they are also the main factor that deters people from using public transportation. The authors’ findings imply that smart transit users may value travel direction in addition to travel time, which influences their mode choice. Depending on their relative location, travelers may prefer even adjacent transfer locations. The authors’ findings will help improve our understanding of transit user behavior and the impact of transfer smartness, as well as smart transportation planning and the design of new transit routes and services to improve transfer performance. This work contributes to the second dimension (data processing) of smartness.

Alomari et al. [6] introduce their tool, Iktishaf+, which combines Big Data, Distributed Machine Learning, social media analytics, and an automatic data labeling method to detect road traffic-related events. It uses a range of technologies, including Apache Spark, Parquet, and MongoDB. The tool can detect and validate several real-world events in Saudi Arabia, including a fire in Jeddah, rain in Makkah, and an accident in Riyadh, without prior knowledge. The findings demonstrate the effectiveness of Twitter media in detecting important events when no prior knowledge of them is available. This work contributed to the first two dimensions of smartness, sensing, and data processing.

Motivated by the fact that over a billion people are disabled worldwide, with 253 million of them being visually impaired or blind, Busaeed et al. [7] propose an approach for collecting data and predicting objects for environment perception, mobility, and navigation using a LiDAR with a servo motor and an ultrasonic sensor. The authors use this approach with a pair of smart glasses called LidSonic V2.0 to help the visually impaired identify obstacles. The LidSonic system consists of a smart glasses-integrated Arduino Uno edge computing device and a smartphone app that transmits data via Bluetooth. Arduino collects data, controls the smart glasses’ sensors, detects obstacles with simple data processing, and provides buzzer feedback to visually impaired users. This work is related to indoor and outdoor mobility, and hence we group this with mobility and transportation research. This work contributes to the first two dimensions of smartness, namely, sensing, and data processing, and moreover, it touches upon the fourth dimension as it discusses its potential to be extended to Cloud and Edge Computing.

## 3. Industry 4.0

The detection of anomalies in harsh industrial environments is a challenging task. To address this, Ghazal et al. [8] propose an Edge–Fog–Cloud architecture, based on mobile IoT edge nodes carried by autonomous robots, for detecting thermal anomalies in aluminum plants. The authors use companion drones as fog nodes and a cloud back-end to analyze thermal anomalies. Moreover, the authors propose a self-driving, deep learning architecture and a thermal anomaly detection and visualization algorithm. Their results show that the proposed robot surveyors are less expensive, have a shorter response time, and detect anomalies more accurately than human surveyors or fixed IoT nodes monitoring the same industrial area. This work contributes to the first, second, and fourth dimensions of smartness, i.e., sensing, data processing, and computing infrastructure.

Campero-Jurado et al. [9] discuss the role of information and communication technologies (ICTs) in advancing occupational health and safety and increasing worker security. Personal Protective Equipment (PPE) based on ICTs reduces the risk of workplace accidents due to the equipment’s ability to make decisions based on environmental factors. Paradigms such as the Industrial Internet of Things (IIoT) and Artificial Intelligence (AI) enable the generation of PPE models and the development of devices with more advanced capabilities such as monitoring, sensing the environment, and risk detection, among others. These models continuously monitor the working environment and notify employees and supervisors of any anomalies or threats. With this context, they propose a smart helmet prototype that monitors the working environment and performs a near real-time risk assessment. The sensor data are sent to an AI-powered platform for analysis. A comparative study of supervised learning models is carried out as part of this research. Furthermore, the use of a Deep Convolutional Neural Network (ConvNet/CNN) is proposed for the detection of potential occupational risks. This work contributes to the first and second dimensions of smartness, namely, sensing and data processing.

## 4. Healthcare

Janbi et al. [10] propose, implement, and evaluate Imtidad, a reference architecture that provides Distributed Artificial Intelligence (AI) as a Service (DAIaaS) over the cloud, fog, and edge. For this purpose, the authors develop a service catalog case study containing 22 AI skin disease diagnosis services. The services are divided into four service classes based on software platforms and are run on a variety of hardware platforms as well as four network types. Two standard Deep Neural Networks (DNNs) and two Tiny AI deep models were trained and tested using real-life dermatoscopic images to create the AI models for diagnosis. Several benchmarks were used to evaluate the services, including model service value, response time, energy consumption, and network transfer time. The services are intended to enable a variety of use cases, such as home patient diagnosis or sending diagnosis requests to traveling medical professionals via a fog device or cloud. This work contributed to the first, second, and fourth dimensions of smartness, sensing, data processing, and computing infrastructure.

## 5. Urban Infrastructure

Yigitcanlar et al. [11] investigate whether artificially intelligent cities can protect humanity from natural disasters, pandemics, and other disasters. By charting the evolution of AI and the potential impacts of systematic AI adoption on cities and societies, the authors generate insights and identify prospective research questions. This viewpoint provides theoretical contributions to all four dimensions of smartness and puts forward directions for future research, as well as listing large number of critical research questions that need to be answered. In another work, Yigitcanlar et al. [12] derive insights from sensor city best practices by scrutinizing some well-known projects implemented by Huawei, Cisco, Google, Ericsson, Microsoft, and Alibaba. The authors highlight that platform urbanism is becoming a critical tool to support smart urban governance in an era of digitalization of urban services and processes. On the basis of the lessons learned from the best practices of leading innovation and technology companies, the study advocates the need for further research on the conceptualization and practice of the sensor city notion.

Feri et al. [13] propose a three-dimensional microstructure reconstruction framework based on a 3D improved Wasserstein Generative Adversarial Network (3D-IWGAN) with an enhanced gradient penalty. It is a computational system based on images for analyzing clogging in the permeable pavement. The physical property values extracted from their model are comparable to those obtained from real pavement samples. The authors are motivated by the fact that there is an increasing demand for research into how to improve the functionality of permeable pavement. Their proposed system starts with a two-dimensional image as input and extracts latent features from it. It generates a 3D microstructure image using their model’s generative adversarial network. This work contributes to the data processing dimension of smartness.

Akram et al. [14] propose an architecture for designing and developing a customized sensor node and gateway based on LoRa (Long-Range radio) technology for solid waste management, specifically, to achieve the filling level of the bins while using the least amount of energy. The authors also include distinct evaluation metrics for the sensor node’s long-range data rate, time on-air (ToA), LoRa sensitivity, link budget, and battery life. LoRa is a popular communication protocol that provides long-range transmission and low data rates while consuming little power. Only a small amount of data needs to be sent to the remote server in the context of solid waste management, hence the use of LoRa. This work contributes to the sensing dimension of smartness.

Jo et al. [15] examined the changes in particulate matter concentrations due to land use over time, as well as the spatial characteristics of the distribution of particulate matter concentrations in Daejeon, Korea, as measured by Private Air Quality Monitoring Smart Sensors (PAQMSSs). According to the primary land use around the 650 m sensor radius, land uses were classified into residential, commercial, industrial, and green groups. The results show that particulate matter concentrations in Daejeon decreased in the order of industrial, housing, commercial, and green groups overall; however, the concentrations of the commercial group were higher than those of the residential group between 21:00 and 23:00, reflecting the commercial group’s vital night-time lifestyle in Korea. The study contributes to the data processing dimension of smartness. Janbi et al. [16] propose a framework for Distributed AI as a Service (DAIaaS) provisioning for the Internet of Everything and 6G environments with the aim to help standardize the mass production of technologies for smarter environments. To investigate the design choices and performance bottlenecks of DAIaaS, multiple DAIaaS provisioning configurations for distributed training and inference are proposed, including three case studies (a smart airport, a smart district, and distributed AI provisioning) with eight scenarios, nine applications and AI delivery models, and 50 distinct sensor and software modules. This work contributes to all four smartness dimensions.

## 6. Concluding Remarks

The smartness that underpins smart cities and societies is defined by our ability to engage with our environments, analyze them, and make decisions, all in a timely manner. The IoT has been the focus of this Special Issue, and its concern has been to bring “smartness” to the IoT and other system layers using emerging technologies. The articles included in this issue cover a wide range of applications, including image analysis, permeable pavements, solid waste management, air quality monitoring, thermal anomalies and smart helmets in industrial environments, smart airports, smart districts, and smart travel choices.

The field of smartness is exciting, and while a lot has been achieved, the future possibilities with technologies such as Deep Learning, Edge Computing, Virtual Reality, and more are endless. There are many works that are complementary to the research presented in this Special Issue, such as deep journalism [17], smartization [18], smart families and homes [19], data-driven smart governance [20,21], responsible innovation [22], and green AI [23]. This is an exciting time for disruptive technologies, and this Special Issue is expected to clarify the concept of smartness, helping more researchers to contribute to this area and lead to the development of truly smart environments.
